# Amyloid precursor protein processing in human neurons with an allelic series of the *PSEN1* intron 4 deletion mutation and total presenilin-1 knockout

**DOI:** 10.1093/braincomms/fcz024

**Published:** 2019-10-14

**Authors:** Charles Arber, Claudio Villegas-Llerena, Jamie Toombs, Jennifer M Pocock, Natalie S Ryan, Nick C Fox, Henrik Zetterberg, John Hardy, Selina Wray

**Affiliations:** 1 Department of Neurodegenerative Disease, UCL Queen Square Institute of Neurology, London WC1N 1PJ, UK; 2 Department of Neuroinflammation, UCL Queen Square Institute of Neurology, London WC1N 1PJ, UK; 3 UK Dementia Research Institute at UCL, London, UK; 4 Department of Neurodegenerative Disease, Dementia Research Centre, UCL Queen Square Institute of Neurology, London WC1N 1PJ, UK; 5 Department of Psychiatry and Neurochemistry, Institute of Neuroscience and Physiology, The Sahlgrenska Academy, University of Gothenburg, Mölndal, Sweden; 6 Clinical Neurochemistry Laboratory, Sahlgrenska University Hospital, Mölndal, Sweden

**Keywords:** Alzheimer’s disease, iPSCs, CRISPR/Cas9, amyloid beta

## Abstract

Mutations in presenilin-1 (*PSEN1*), encoding the catalytic subunit of the amyloid precursor protein-processing enzyme γ-secretase, cause familial Alzheimer’s disease. However, the mechanism of disease is yet to be fully understood and it remains contentious whether mutations exert their effects predominantly through gain or loss of function. To address this question, we generated an isogenic allelic series for the *PSEN1* mutation intron 4 deletion; represented by control, heterozygous and homozygous mutant induced pluripotent stem cells in addition to a presenilin-1 knockout line. Induced pluripotent stem cell-derived cortical neurons reveal reduced, yet detectable amyloid-beta levels in the presenilin-1 knockout line, and a mutant gene dosage-dependent defect in amyloid precursor protein processing in *PSEN1* intron 4 deletion lines, consistent with reduced processivity of γ-secretase. The different effects of presenilin-1 knockout and the *PSEN1* intron 4 deletion mutation on amyloid precursor protein-C99 fragment accumulation, nicastrin maturation and amyloid-beta peptide generation support distinct consequences of familial Alzheimer’s disease-associated mutations and knockout of presenilin-1 on the function of γ-secretase.

## Teaser

We report the generation of an allelic, isogenic series for the familial Alzheimer’s disease mutation *PSEN1* intron 4 deletion as well as total presenilin-1 knockout. Amyloid-beta profiles follow a mutation dose-dependent pattern. Presenilin-1 knockout lines display distinct phenotypes from intron 4 deletion lines, including reduced mature nicastrin, arguing against a simple loss-of-function mechanism for familial Alzheimer’s disease mutations.

## Graphical Abstract

**Graphical Abstract:**
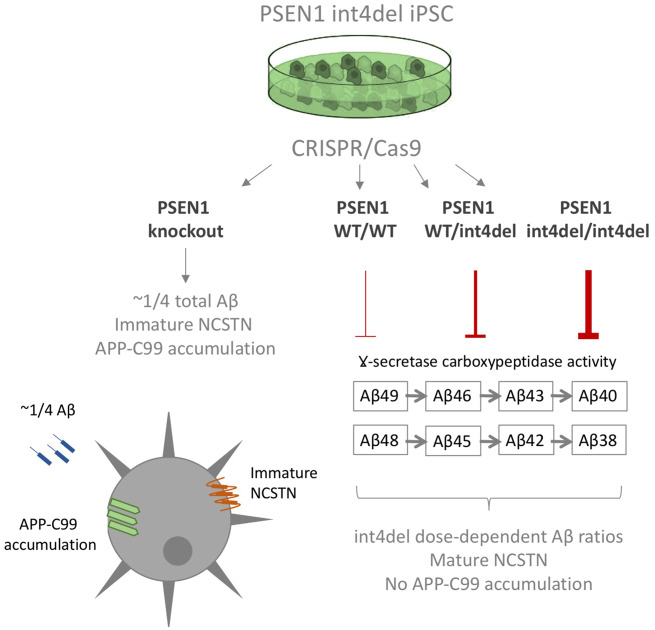

## Introduction

Amyloid precursor protein (APP) is cleaved by γ-secretase, the catalytic subunit of which consists of presenilin-1 (PSEN1) or presenilin-2 (PSEN2), to produce amyloid β (Aβ). Mutations in *APP* and *PSEN1/2* that cause familial Alzheimer’s disease (fAD) are believed to alter this interaction, increasing the relative proportion of aggregation-prone Aβ species (Ryan *et al.*, 2016), and forming the basis of the amyloid cascade hypothesis (Hardy and Allsop, 1991).

PSEN1 and PSEN2 are alternate catalytic subunits of γ-secretase, a tetrameric protein complex also containing nicastrin (NCSTN), PSEN enhancer (PEN2) and alternate subunits anterior pharynx 1a/b (APH1a/b; De Strooper, 2003). γ-Secretase serves as a membrane protease, cleaving numerous substrates (Haapasalo and Kovacs, 2011) that include the products of β-secretase and α-secretase cleavage of APP (C99 and C83, respectively). Cleavage of C99 by γ-secretase occurs through an initial endopeptidase-like activity followed by subsequent carboxypeptidase-like cleavages to generate shorter Aβ fragments (Takami *et al.*, 2009; Matsumura *et al.*, 2014). In addition, γ-secretase-independent activities for PSEN1 have been described, such as a chaperone activity crucial for the glycosylation and maturation of NCSTN (Leem *et al.*, 2002).

*PSEN1* mutations have been shown to consistently reduce the carboxypeptidase-like activity of γ-secretase, leading to the accumulation of longer, more amyloidogenic Aβ species, such as Aβ42 and Aβ43 (Chávez-Gutiérrez *et al.*, 2012; Szaruga *et al.*, 2015; Arber *et al.*, 2019). This can be evidenced as an increased Aβ42:38 ratio (Takami *et al.*, 2009; Matsumura *et al.*, 2014). The *PSEN1* intron 4 deletion mutation (L113_I114insT; hereafter referred to as int4del) describes the deletion of a guanine nucleotide in the splice donor region of *PSEN1* after exon 4 leading to three alternative transcripts; one coding a full-length protein with an insertion of an additional threonine in the PSEN1 protein, and two shorter transcripts with premature stop codons (De Jonghe *et al.*, 1999). The long transcript was shown to be responsible for elevated Aβ42 generation (De Jonghe *et al.*, 1999). We and others have previously shown that this mutation increases the Aβ42:38 ratio in patient-derived iPSC-neurons and potentially reduces overall γ-secretase activity (Moore *et al.*, 2015; Arber *et al.*, 2019).

There has been contention over the question of whether mutant *PSEN1* alleles confer predominantly gain or loss of function (Veugelen *et al.*, 2016; Xia *et al.*, 2016). In order to further investigate the molecular mechanisms of the *PSEN1* int4del mutation in a human neuronal system, we used CRISPR/Cas9 gene editing to produce an isogenic allelic series from patient-derived iPSCs. The series is represented by isogenic control (wild type) cells, heterozygous and homozygous mutation-bearing cells, as well as PSEN1 knockout cells. We find that iPSC-derived cortical neurons maintain Aβ generation in PSEN1 knockout cells and display a mutant gene dosage-dependent phenotype on APP/Aβ processing and Aβ42 generation. The data support distinct effects of fAD-associated mutations and PSEN1 protein knockout.

## Materials and methods

### Cell culture

The acquisition of patient fibroblasts for the generation of iPSC was approved by the National Hospital for Neurology and Neurosurgery and Institute of Neurology Joint Research Ethics Committee (Study Title: Induced pluripotent stem cells derived from patients with familial Alzheimer’s disease and other dementias as novel cell models for neurodegeneration, Reference: 09/H0716/64).

All reagents are from ThermoFisher unless specified. *PSEN1* int4del iPSCs were obtained from StemBancc and cultured in Essential 8 media on Geltrex substrate and passaged using 0.5 mM EDTA, with the exception of gene editing steps that were performed in mTESR media (Stem Cell Technologies). Differentiation was performed following published protocols (Shi *et al.*, 2012). In brief, iPSCs were grown to confluency and switched to N2B27 media containing 10 µM SB431542 and 1 µM dorsomorphin (both Tocris). N2B27 media is composed of a mix of 1:1 DMEM-F12 and Neurobasal supplemented with 0.5× N2 supplement, 0.5× B27 supplement, 0.5× non-essential amino acids, 1 mM l-glutamine, 25 U pen/strep, 10 µM β-mercaptoethanol and 25 U insulin. Following 10 days of neural induction, cells were maintained in N2B27 without SB431542 and dorsomorphin until day 100, which was taken as the final time point for neuronal analysis. DAPT (Tocris) treatment was performed with 10 µM DAPT for 48 h.

### Karyotype analysis

Genomic DNA from iPSC lines was tested using the hPSC Genetic Analysis Test (Stem Cell Technologies). Analysis of the eight most common PSC mutation sites showed there were no significant chromosomal abnormalities in the four iPSC lines. Results highlight possible, non-significant abnormalities that are believed to be PCR artefacts.

### CRISPR/Cas9-mediated genome editing

sgRNAs were designed and constructed using the CRISPR finder tool from the WTSI Genome Editing (WGE) website (Hodgkins *et al.*, 2015). The single-stranded donor oligonucleotide (ssODN, Table 1), used as homologous recombinant template, was designed to correct the int4del mutation in the heterozygous patient cells. ssODN was asymmetric (60 and 35 bp to the 5ʹ and 3ʹ ends of the double-strand break, respectively) and was synthesized with phosphorothioate modifications (IDT) to increase the efficiency of homology-directed DNA repair (Renaud *et al.*, 2016). For CRISPR/Cas9 gene editing, we used the RNP (ribonucleoprotein) delivery strategy. RNP preparation was performed as per the manufacturer’s protocol using the Alt-R™ CRISPR tracrRNA, crRNA and S.p. Cas9 Nuclease 3NLS (IDT). Both the ssODN and the RNP were delivered simultaneously into patient-derived iPSCs by electroporation (Amaxa 4D, Lonza). Transfected cells were seeded and expanded in mTesr media + 10 µm ROCK inhibitor (Stem Cell Technologies). Screening of modified clones was performed by restriction fragment length polymorphism (RFLP) using the RsaI enzyme, which specifically cleaves the WT and not the int4del pathogenic allele. Edited clones were confirmed by polymerase chain reaction (PCR) and Sanger sequencing. Off-target sites were predicted using the CRISPR finder tool and the sgRNA sequence used for gene editing. PCR primers were designed for the amplification of the top-5 potential off-target sites (Table 1). Sanger sequencing screening of our isogenic allelic series of iPSC cells showed no evidence of off-target activity at any of the screened loci. Sequence alignment was performed using SnapGene software and chromatograms with frame shift mutations were deconvoluted using the Indigo programme (gear.embl.de/indigo/).

**Table 1:** Primers used in this study

	Assay	Forward	Reverse
PSEN1 int4del specific	PCR (gDNA)	CGGAAGGATGGGCAGCTTACA	AGCCACGCAGTCCATTCAGG
PSEN1 exon 4 splicing	PCR (cDNA)	TGAGGACAACCACCTGAGCAA	TGGCAGCATTCAGAATTGAGT
PSEN1	Sequencing/RFLP	AGGTCTAACCGTTACCTTGATTC	CAGCCCTATCCAGTAATACCATAC
ssODN	CRISPR/Cas9	T*C*TCTGCATGGTGGTGGTCGTGGCTACCATTAAGTCAGTCAGCTTTTATACCCGGAAGGATGGGCAGCTGTACGTATGAGTTTTGTTTTATTA*T*T	
sgRNA	CRISPR/Cas9	AGCTTTTATACCCGGAAGGA	
Off-target 1	Sequencing	CACAGCGTTCACGTTGTATTG	CCAGGGAAGAAACAGAGACTAAC
Off-target 2	Sequencing	ACAAGTAGACTTCTAGGCTGAAAC	CATGACTTCCTGAGGAGAACAG
Off-target 3	Sequencing	TAGGTAAACACTGGCTGGAAAG	AGCAAGTGGGAAAGAAGACC
Off-target 4	Sequencing	GCCTGCGATTTGAGGGATAAC	AGTTAAAGGGAGCAGGGACTAC
Off-target 5	Sequencing	GAATCCTCCAGCCGGTCTTC	CAGCCCTGTCCCACCTTTC
*RPL18a*	qPCR	CCCACAACATGTACCGGGAA	TCTTGGAGTCGTGGAACTGC
*APP*	qPCR	GGTACCCACTGATGGTAAT	GGTAGACTTCTTGGCAATAC
*PSEN1*	qPCR	TATCAAGTACCTCCCTGAAT	ACCATTGTTGAGGAGTAAAT
*PSEN2*	qPCR	ATCTCTGTGTATGATCTCGT	TCCCCAAAACTGTCATAG
*BACE1*	qPCR	GTCTCTGGTATACACCCATC	CATAGTTGTACTCCTTGCAG
*TUBB3*	qPCR	CATGGACAGTGTCCGCTCAG	CAGGCAGTCGCAGTTTTCAC
*TBR1*	qPCR	AGCAGCAAGATCAAAAGTGAGC	ATCCACAGACCCCCTCACTAG
*CTIP2*	qPCR	CTCCGAGCTCAGGAAAGTGTC	TCATCTTTACCTGCAATGTTCTCC
*ECE1*	qPCR	AGTGACACAGAAAACAACCT	GAACTGCAGTGTAGTCATTAAA
*ACE*	qPCR	GAAGTTTGATGTGAACCAGT	ACAGGATCTTGTTGTACTCCT
*IDE*	qPCR	CAAAGACTCACTCAACGAG	CTGAAAGATACATCCCATAG
*NEP*	qPCR	GAGGGGTCACGATTTTAG	AAGTCTGTACAAGGCTCAGT

* Phosphorothioate modification.

### Immunocytochemistry

Cells were fixed for 15 min in 4% paraformaldehyde. Following fixation, cells were washed three times in PBS with 0.3% Triton-X-100 (PBST) and blocked in 3% bovine serum albumin in PBST for 20 min. Primary antibodies (Table 2) were added to cells overnight at 4°C in blocking solution. Cells were washed three times in PBST and Alexafluor secondary antibodies were added in blocking solution for 1 h at room temperature. Following three final washes in PBST (the first of which contained DAPI nuclear counterstain), cells were mounted in fluorescence mounting media (DAKO) and imaged on a Zeiss LSM microscope.

**Table 2:** Antibodies used in this study

Antibody	Company	Species
SSEA4	Biolegend MC-813–70	Mouse
OCT4	SantaCruz sc5297	Goat
TUJ1	Biolegend 801201 and 802001	Mouse and rabbit
TBR1	Abcam ab31940	Rabbit
APP 6e10	Biolegend 803014	Mouse
APP C-term	Biolegend 802803	Mouse
PSEN1 N-term	Millipore MAB1563	Rat
PSEN1 C-term	Millipore MAB5232	Mouse
PSEN2	Cell Signaling Technologies 9979	Rabbit
NCSTN	BD Transduction Labs 612290	Mouse
Actin	Sigma 1978	Mouse

### Polymerase chain reaction

RNA was isolated from neurons using Trizol reagent following the manufacturer’s protocols. cDNA was generated using superscript IV following the manufacturers’ protocols with 2 µg of total RNA and using random hexamer primers. PCR was performed using GoTaq PCR mastermix (Promega). Quantitative PCR (qPCR) was performed using Power SYBR green and an MX3000P thermocycler (Agilent). Primers are shown in Table 1.

### Western blotting

Cells were lysed in RIPA buffer containing protease and phosphatase inhibitors (Roche). Lysates were denatured in NuPage LDS buffer and loaded onto 4–12% precast polyacrylamide gels in MES running buffer (NuPage/ThermoFisher). Proteins were transferred to a nitrocellulose membrane, blocked in 3% bovine serum albumin and blotted using antibodies in Table 2. Images were taken and analysed on an Odyssey Fc (LiCor Biosciences).

### Aβ-Electrochemiluminescence

Forty-eight hours conditioned media were collected from neuronal cultures and centrifuged to remove cell debris. Aβ42, Aβ40 and Aβ38 were quantified simultaneously using the Meso Scale Discovery V-Plex Aβ peptide panel kit (6E10) by electrochemiluminescence. Samples were diluted 1:1 with diluent 35 and measurements were made on the MSD Sector 6000. Aβ concentrations in the cell media were normalized to cell pellet protein concentration, measured using BioRad BCA assay.

### Statistical analysis

Data analysis was performed in Microsoft Excel and GraphPad Prism 7. Samples were compared via one-way ANOVA with subsequent *post* *hoc* Tukey’s multiple comparisons test (*P > 0.05, **P > 0.01, ***P > 0.001, ****P > 0.0001). Error bars on histograms show ± standard deviation of the mean and independent experimental replicates are shown via numbers within histograms.

### Data availability

The authors confirm that all the data supporting the findings of this study are available within the article and readily available upon request. For ANOVA analyses, exact *P*-values, *F*-values and degrees of freedom are presented in the Supplementary material.

## Results

### Generation of *PSEN1* int4del allelic series

**Figure 1:**
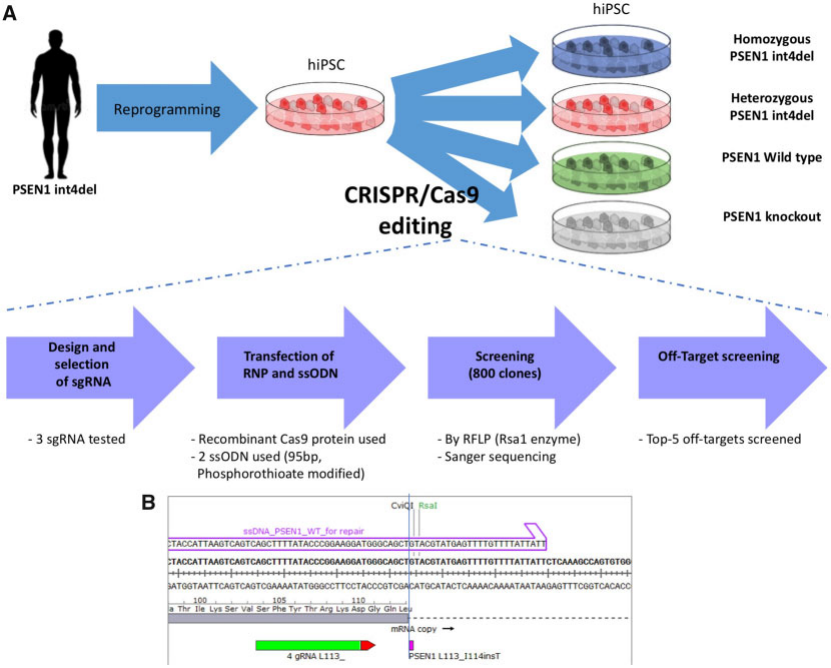
**Scheme for the gene editing of patient-derived *PSEN1* int4del iPSCs.** (**A**) Strategy for the generation of isogenic cells using CRISPR/Cas9-editing of an iPSC line from an individual carrying the *PSEN1* int4del mutation. (**B**) Genomic positioning of the editing site, showing ssODN repair arm (purple), mutation site (purple) and sgRNA (green) with PAM site (red). sgRNA (single guide RNA, for CRISPR/Cas9 targeting); RFLP (used for screening); ssODN (single-stranded oligodeoxynucleotide, for homology-directed repair).

CRISPR/Cas9 gene editing was used to generate an isogenic series of iPSC lines from a patient-derived *PSEN1* int4del iPSC line (Fig. 1). In order to generate an allelic series, a PAM site 6 base pairs upstream of the mutation was selected, recognizing both mutant and wild-type alleles. This enables both homology-directed repair from the ssODN (single-stranded oligodeoxynucleotide) and template-free repair of the pathogenic variant in the same CRISPR/Cas9 transfection (Shen *et al.*, 2018). For increased efficiency of homology-directed DNA repair, the ssODN template was modified to contain phosphorothioate moieties (Renaud *et al.*, 2016).

**Figure 2:**
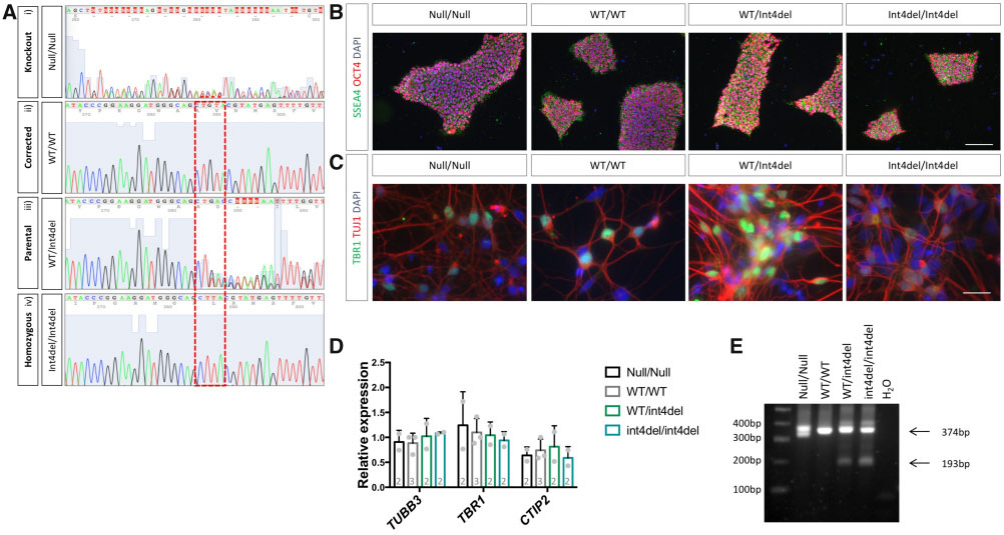
**Characterization of gene-edited iPSC and neurons.** (**A**) Sanger sequencing was used to confirm the generation of a PSEN1 knockout (i), a corrected wild-type *PSEN1* line (ii), an unedited heterozygous mutant line (iii) and a homozygous *PSEN1* int4del mutation line (iv). (**B**) Immunocytochemistry was performed on all iPSC lines following CRISPR/Cas9 editing to confirm pluripotency with the pluripotency markers OCT4 (red) and SSEA4 (green). Scale bar 100 μm. (**C**) Successful differentiation of iPSC into neurons was characterized 50 days post-induction by immunocytochemistry for the neuronal marker TUJ1 (red) and deep-layer cortical neuronal marker TBR1 (green). Scale bar 25μm. (**D**) Further characterization of cortical differentiation was performed using qPCR analysis 100 days post-neural induction to assess expression of neuronal marker *TUBB3* and cortical layer markers *TBR1* and *CTIP2*. Numbers within histogram represent the number of independent neural inductions. (**E**) PCR analysis of *PSEN1* splicing in cDNA from day 100 neurons using primers recognizing exons 3 and 5 of *PSEN1* mRNA. The full-length transcript is depicted at 374 bp and one short transcript caused by aberrant splicing is evident at 193 bp in mutation-bearing neurons (we do not see evidence of a second aberrantly spliced band at 111 bp).

Following an initial screen of 800 iPSC colonies by RFLP (see Materials and methods section), Sanger sequencing was used to confirm the generation of; (i) an isogenic control cell line, (ii) an unedited line, (iii) a homozygous int4del line and (iv) a PSEN1 knockout line (Fig. 2A). The knockout line was a compound heterozygous, which contained a 4 and a 25 base pair deletion; each leading to a reading frame shift (Supplementary Fig. 1). The allelic series was screened and found to be free from off-target nucleotide changes at five most likely genomic sites (see Materials and methods section) and pluripotency was confirmed via the expression of OCT4 and SSEA4 (Fig. 2B). Karyotype stability was tested and no significant aberrations were found (Supplementary Fig. 2). iPSCs were subjected to cortical differentiation, generating the cell type affected by fAD (Shi *et al.*, 2012). All lines generated neurons with a similar efficiency, as evidenced by the expression of the deep-layer cortical marker TBR1 by immunocytochemistry and the population expression level of *TUBB3*, *TBR1* and *CTIP2* by qPCR (Fig. 2C and D). Finally, to confirm the mutation status of the iPSC-derived neurons, cDNA was analysed by PCR to depict aberrant splicing of *PSEN1* in mutation-bearing neurons (Fig. 2E; Tysoe *et al.*, 1998). In addition to the full-length L113_I114insT encoding transcript (374 bp), heterozygous and homozygous *PSEN1* int4del lines show evidence of one short transcript produced by aberrant splicing (193 bp).

### iPSC-derived PSEN1 knockout neurons display aberrant APP processing

**Figure 3:**
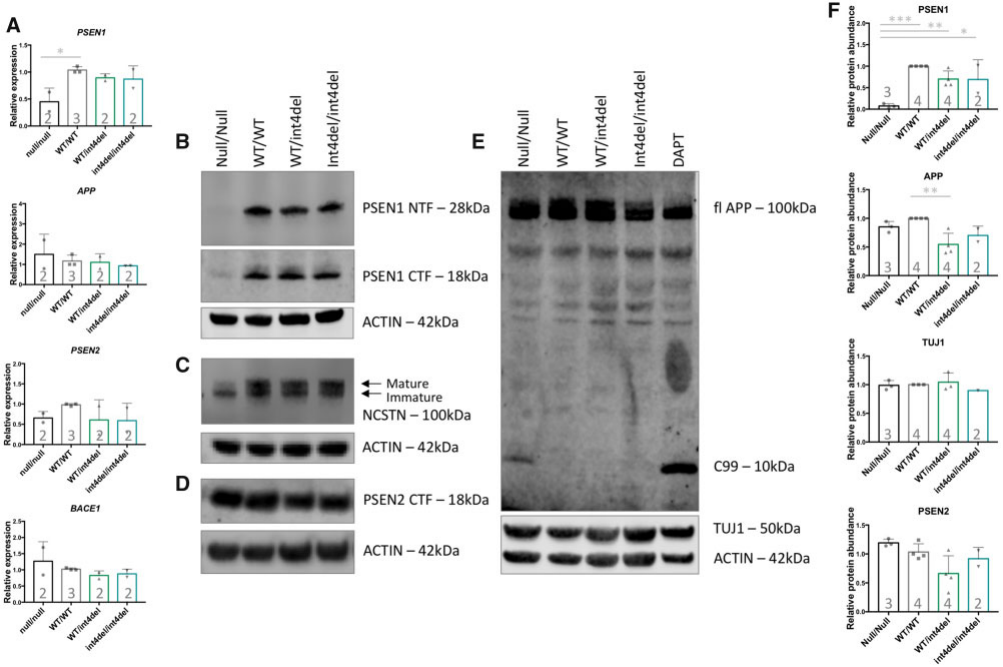
**Analysis of γ-secretase and APP processing in iPSC-derived neurons.** (**A**) *PSEN1*, *PSEN2*, *APP* and *BACE1* expression in iPSC-derived neurons 100 days post-induction was assessed by qPCR in neurons from PSEN1 knockout, *PSEN1* wild type, *PSEN1* int4del heterozygous and *PSEN1* int4del homozygous lines. *PSEN1* expression was significantly reduced in the PSEN1 knockout neurons. No significant differences in *PSEN2*, *APP* and *BACE1* were observed. (**B–E**) Western blot on whole cell lysates of day 100 neurons was used to analyse protein levels of PSEN1 N-terminal fragments (28 kDa), PSEN1 C-terminal fragments (18 kDa), NCSTN (100 kDa), PSEN2 C-terminal fragments (18 kDa), APP (100 kDa) and neuronal marker TUJ1 (50 kDa). The DAPT sample represents an unrelated control line treated with the γ-secretase inhibitor DAPT at 10 μM for 48 h. (**F**) Quantification of western blot band intensities in **B–E**. PSEN1 protein abundance is significantly reduced in *PSEN1* knockout lysates and APP is significantly increased in the corrected wild-type neurons compared with parental *PSEN1* int4del. *P > 0.05, **P > 0.01, ***P > 0.001 by one-way ANOVA with Tukey’s *post hoc* analysis. Numbers within histograms represent the number of independent neural inductions.

Using primers 3ʹ to the site of gene editing, qPCR analysis showed reduced *PSEN1* expression in the knockout cell line (Fig. 3A). Interestingly, *PSEN2* showed similar expression in all cell lines, suggesting that loss of PSEN1 does not result in compensation by up-regulation of this alternate γ-secretase subunit. Expression of *APP* and *BACE1* was not significantly altered based on mutation status.

At the protein level, the expected absence of PSEN1 protein was confirmed in the knockout line (using antibodies that recognize the N- or C-terminus of PSEN1; Fig. 3B). The PSEN1 knockout line also showed a reduced maturation of NCSTN, evidenced as a relative lack of the larger band that represents the glycosylated protein (Leem *et al.*, 2002). This phenotype was not seen in lines containing the mutation (Fig. 3C). Similar to qPCR analysis, there was no apparent compensatory up-regulation of PSEN2 protein in PSEN1 knockout cells as analysed by western blot (Fig. 3D). Total APP levels were largely equivalent (Fig. 3E and F); however, an accumulation of a fragment predicted to be C99 was present in the PSEN1 knockout neurons—analogous to γ-secretase-inhibited wild-type cells (Fig. 3E).

### Aβ is produced in knockout cells and disease-associated processing defects are dependent on mutant gene dosage

**Figure 4:**
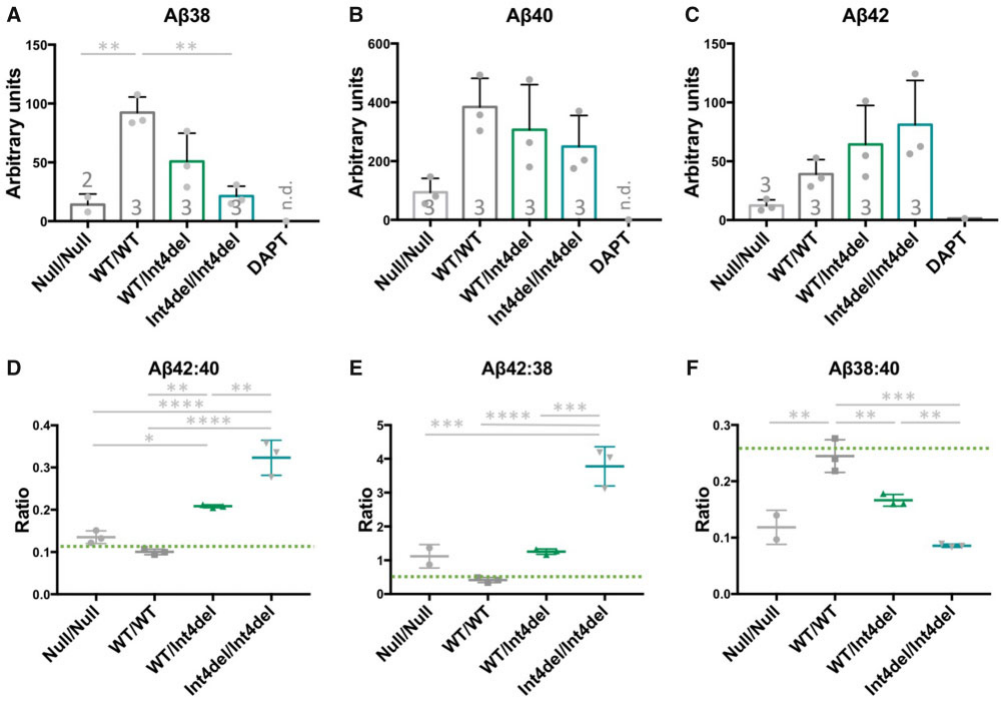
**Aβ analysis in iPSC-neuronal-conditioned media.** (**A–C**) Quantification of Aβ38, Aβ40 and Aβ42 in 48 h-conditioned media from neurons at day 100, measured by electrochemiluminescence and normalized to total protein content from the cell pellet. The DAPT sample is a representative unrelated control line treated with 10 μM of the γ-secretase inhibitor DAPT for 48 h; n.d., not detected. Numbers within histograms represent the number of independent neural inductions. (**D–F**) Aβ ratios to depict the disease-associated Aβ42:40 ratio, γ-secretase carboxypeptidase-like activity Aβ42:38 and endopeptidase cleavage position choice Aβ38:40. *n*=3 for each sample apart from where Aβ38 was below detection limit for one null/null sample. Green bars represent non-Alzheimer’s disease neuronal ratios from Arber *et al.* (2019). *P > 0.05, **P > 0.01, ***P > 0.001 and ****P > 0.0001 by one-way ANOVA with Tukey’s *post hoc* analysis.

To investigate the molecular effects of the *PSEN1* int4del mutation, we analysed the Aβ profiles of the neuronal condition media 100 days post neural induction. PSEN1 knockout cells produced less Aβ compared with other lines, reaching significance for Aβ38 (Fig. 4A–C). The levels of Aβ remained within detection limits, which are in contrast to neurons treated with the γ-secretase inhibitor DAPT, where Aβ was at or below the detection threshold. Expression of four Aβ degrading enzymes remains consistent between genotypes (Supplementary Fig. 3), suggesting that the detection of Aβ is not a result of reduced degradation.

Knockout cells produced a non-significant increase in the Aβ42:40 and Aβ42:38 ratios and a significantly reduced Aβ38:40 ratio when compared with isogenic control cells (Fig. 4D–F). These changes are analogous to the changes witnessed between the wild type and the heterozygous int4del line.

The heterozygous and homozygous int4del lines showed increased total levels of Aβ42 and reduced production of Aβ38 in a gene dosage-dependent manner (Fig. 4A–C). This corresponds to similar stepwise mutant gene dosage-dependent changes to Aβ42:40, Aβ42:38 and Aβ38:40 (Fig. 4D–F). In each instance, the homozygous line was significantly different from the patient-derived heterozygous line.

## Discussion

We successfully generated an isogenic allelic series of the *PSEN1* mutation int4del with which to investigate the molecular mechanisms of fAD mutation-dependent effects on APP processing. We found that PSEN1 knockout cells produce low levels of Aβ and that *PSEN1* int4del heterozygous and homozygous cells produce a stepwise increase in longer, disease-associated Aβ peptides.

It is intriguing that PSEN1 knockout neurons produce small amounts of Aβ. This is in contrast to γ-secretase-inhibited cells where Aβ species are often below the detection limit but in agreement with the detection of around one-fifth of total Aβ levels in mouse PSEN1 knockout primary neurons (De Strooper *et al.*, 1998). It is noteworthy that Aβ is barely detectable after γ-secretase administration to patients during clinical trials (Gillman *et al.*, 2010). We provide evidence for no compensatory up-regulation of PSEN2 at either the transcriptional or protein level, which could potentially be due to alternate subcellular compartmentalization (Sannerud *et al.*, 2016). These data suggest that PSEN2 may produce low levels of Aβ in neurons. Alternatively, the lack of compensation by PSEN2 in *PSEN1* knockout neurons, taken together with the reduced functional γ-secretase pool, seen via NCSTN immaturity, promotes the hypothesis that alternative Aβ-generating enzymes are contributing to Aβ production in PSEN1 knockout human neurons. This hypothesis is reinforced as Aβ is detectable in PSEN1 and PSEN2 double knockout mice (Wilson *et al.*, 2002). It should be noted that in non-neuronal cells, knockout of either PSEN1 or PSEN2 does not alter total Aβ generation, whereas double knockout drastically reduces total Aβ (Lessard *et al.*, 2019). Candidates for C-terminal Aβ degrading activity include matrix metalloproteinase 2 or 9 (MMP2/9; Hernandez-Guillamon *et al.*, 2015) and cathepsin B (Mueller-Steiner *et al.*, 2006).

The finding that PSEN1 knockout cells have a relative increase in Aβ42 compared with Aβ38 means that variability in *PSEN1* expression levels could contribute to altered Aβ profiles in Alzheimer’s disease.

It is important that int4del homozygous cells display an additional, stepwise increase in disease-associated Aβ profiles compared with the patient-derived lines. This equates to a linear increase in total Aβ42 production. Our data demonstrate a mutant gene dosage-dependent effect in Aβ42 generation reinforcing similar findings with the *PSEN1* exon 9 mutation (Woodruff *et al.*, 2013), the *PSEN1* M146I mutation (Paquet *et al.*, 2016) and recently 7 fAD mutations investigated by genome editing (Kwart *et al.*, 2019). The PSEN1 knockout neurons and mutation-bearing neurons show different phenotypes with respect to quantitative production of Aβ and, taken together with the dissimilar effects on C99 accumulation and NCSTN maturation, these data argue against a simple loss-of-function mechanism for *PSEN1* mutations. In agreement with these findings and in contrast to fAD-causing mutations, *PSEN1* loss-of-function mutations have been found to cause acne inversa rather than dementia (Wang *et al.*, 2010).

The fact that *PSEN1* int4del homozygosity does not lead to altered NCSTN maturation, suggests that the effect of the mutation acts on γ-secretase as a whole and not on the activity of PSEN1 itself. This is reinforced by recent crystal structure findings (Yang *et al.*, 2019; Zhou *et al.*, 2019) and mechanistic studies (Szaruga *et al.*, 2017; Petit *et al.*, 2019) whereby fAD mutations appear to destabilize substrate to holo-enzyme complex interaction to release longer Aβ fragments before complete enzymatic processing, rather than altering the PSEN1 subunit activity.

The fact that APP protein is significantly increased in the isogenic control compared with the parental *PSEN1* int4del heterozygous line is intriguing. In our previous work, total APP is not significantly altered in the patient-derived line versus unrelated controls (Arber *et al.*, 2019). We believe the slight increase in APP total levels in the corrected line may directly relate to the correction of the mutation.

In conclusion, data from this isogenic human neuronal allelic series reinforce the findings that fAD-associated mutations in *PSEN1* lead to accumulation of Aβ by reducing the processivity of APP by γ-secretase. Mutations reduce carboxypeptidase-like activity, releasing longer amyloidogenic Aβ peptides in a gene dose-dependent manner. PSEN1 knockout cells generate Aβ and also show distinct substrate processing alterations from int4del homozygous cells, potentially separating γ-secretase-dependent and independent functions of PSEN1 and arguing against a simple loss-of-function mechanism. These findings support a destabilization of γ-secretase-substrate interaction by the mutation; information that is valuable for the design of novel therapeutics.

## Supplementary Material

fcz024_Supplementary_Data

## Abbreviations

β =: amyloid beta
APP =: amyloid precursor protein
CRISPR =: clustered regularly interspersed short palindromic repeats
DAPT =: γ-secretase inhibitor N-[N-(3, 5-Difluorophenacetyl)-L-alanyl]-S-phenylglycine t-butyl ester
fAD =: familial Alzheimer’s disease
iPSC =: induced pluripotent stem cell
NCSTN =: nicastrin
PSEN1/2 =: presenilin-1 or presenilin-2
RFLP =: restriction fragment length polymorphism
sgRNA =: single guide RNA
ssODN =: single-stranded oligodeoxynucleotide
