# Supplementary material for: Amyloid precursor protein processing in human neurons with an allelic series of the PSEN1 intron 4 deletion mutation and total presenilin-1 knockout
Source: Brain Commun. 2019 Oct 14;1(1):fcz024. doi: 10.1093/braincomms/fcz024 (PMC7212081; doi:10.1093/braincomms/fcz024)
Supplement: fcz024_Supplementary_Data [file fcz024_supplementary_data.zip › Supplementary_Figure_Legends.pdf]

Supplementary Figure 1.
A) Depiction of indels in PSEN1 knockout iPSCs. Sanger sequencing shows the line is a compound heterozygous line with 4 and 25 base pair deletions, causing frame shifts in both alleles.
B-C) Uncropped images of western blots shown in Figure 3.

Supplementary Figure 2.
Karyotype analysis of iPSC lines using the hPSC Genetic Analysis Test (Stem Cell Technologies) showing copy number for 8 regions that are commonly prone to chromosomal abnormalities in iPSC cultures. Possible abnormalities are highlighted with red arrows, but are not significant and believed to be technical false positives.

Supplementary Figure 3.
qPCR analysis of four Aβ degrading enzymes (*ECE1, ACE, IDE* and *NEP*). No significant differences were evident in expression levels of these enzymes between *PSEN1* knockout, *PSEN1* wildtype, *PSEN1* int4del heterozygous and *PSEN1* int4del homozygous lines.
