# Supplementary figures and images for: Amyloid precursor protein processing in human neurons with an allelic series of the PSEN1 intron 4 deletion mutation and total presenilin-1 knockout
Source: Brain Commun. 2019 Oct 14;1(1):fcz024. doi: 10.1093/braincomms/fcz024 (PMC7212081; doi:10.1093/braincomms/fcz024)

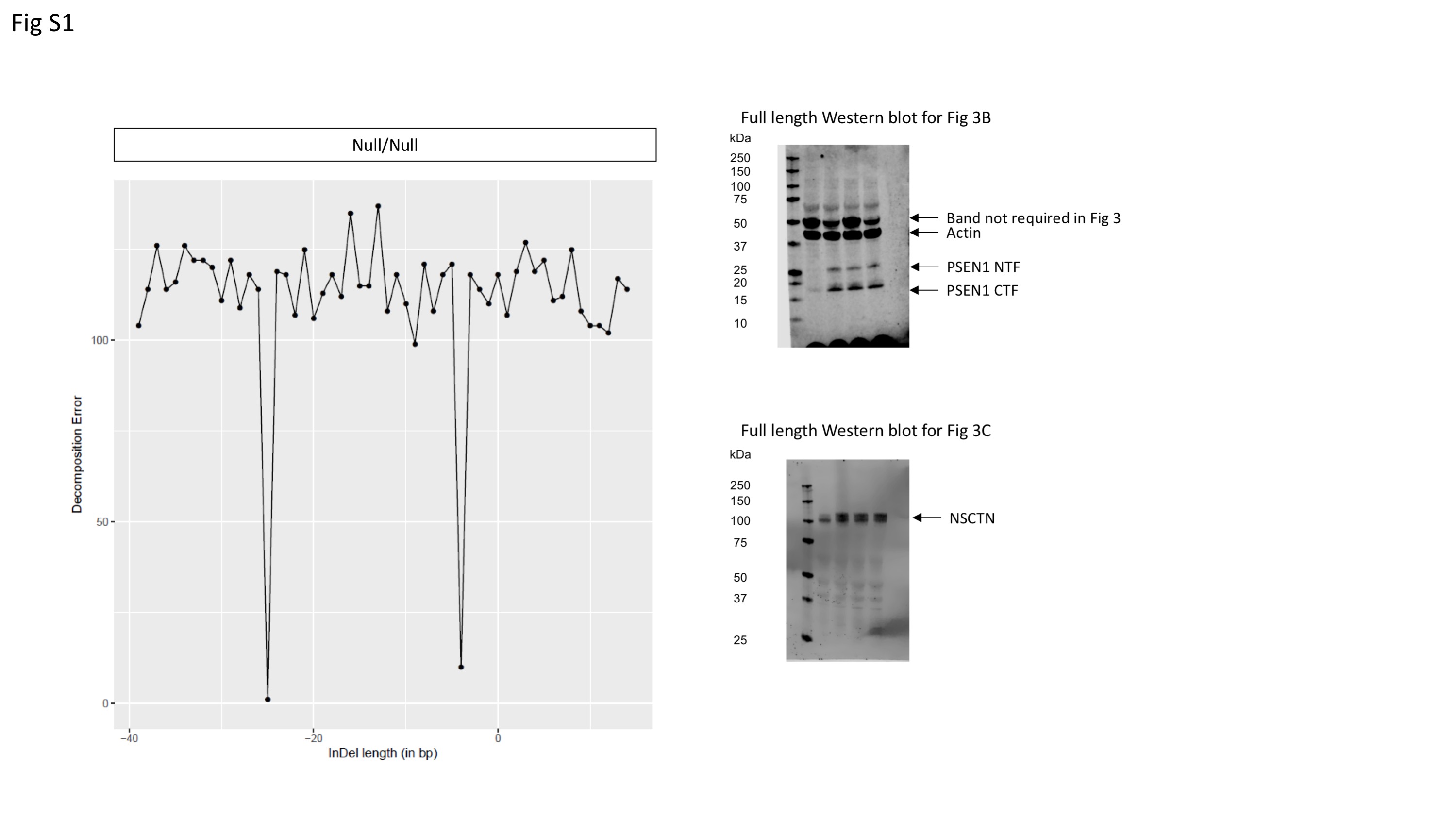

Supplement: fcz024_Supplementary_Data [file fcz024_supplementary_data.zip › Fig S1.jpeg]

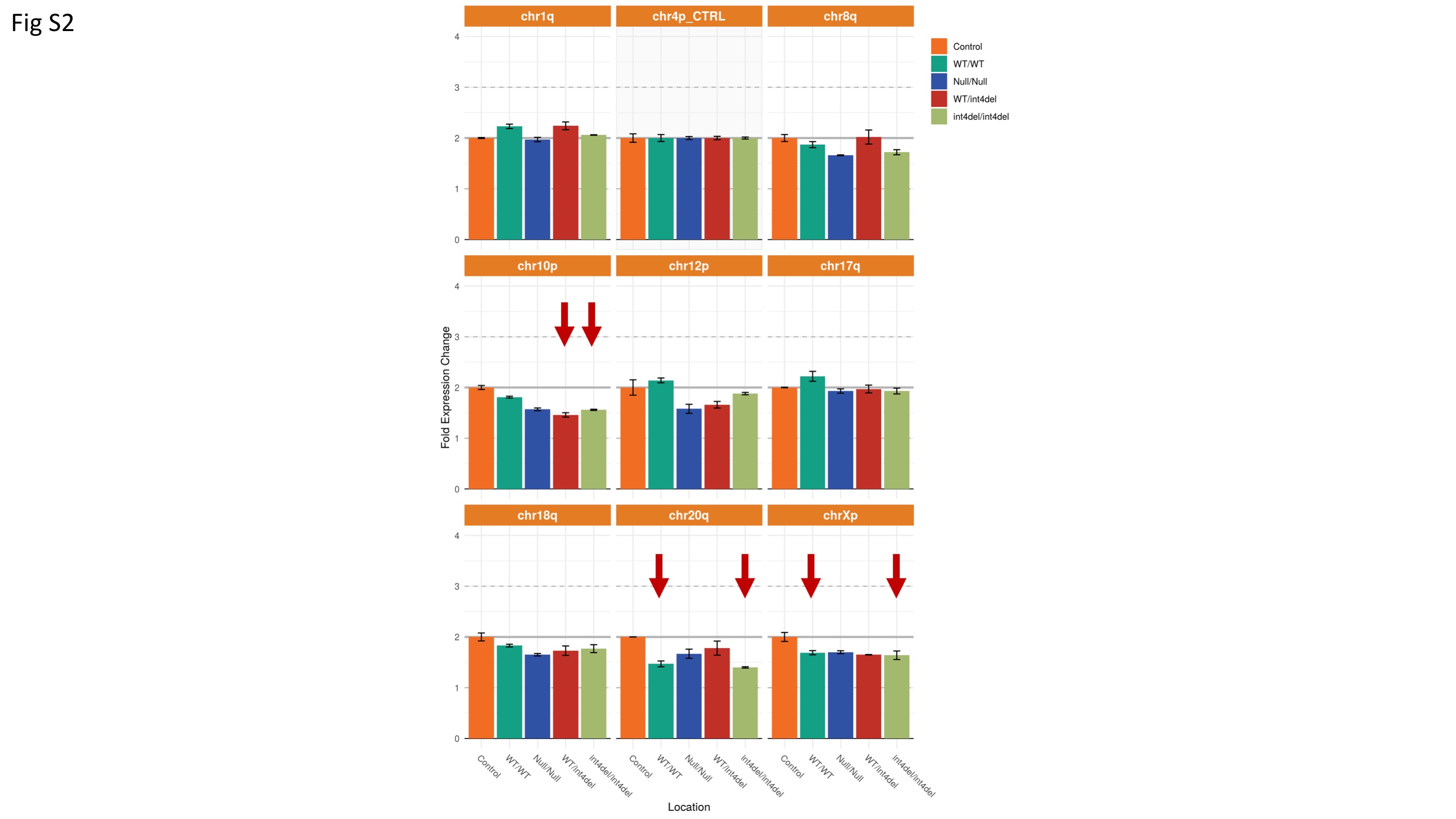

Supplement: fcz024_Supplementary_Data [file fcz024_supplementary_data.zip › Fig S2.jpeg]

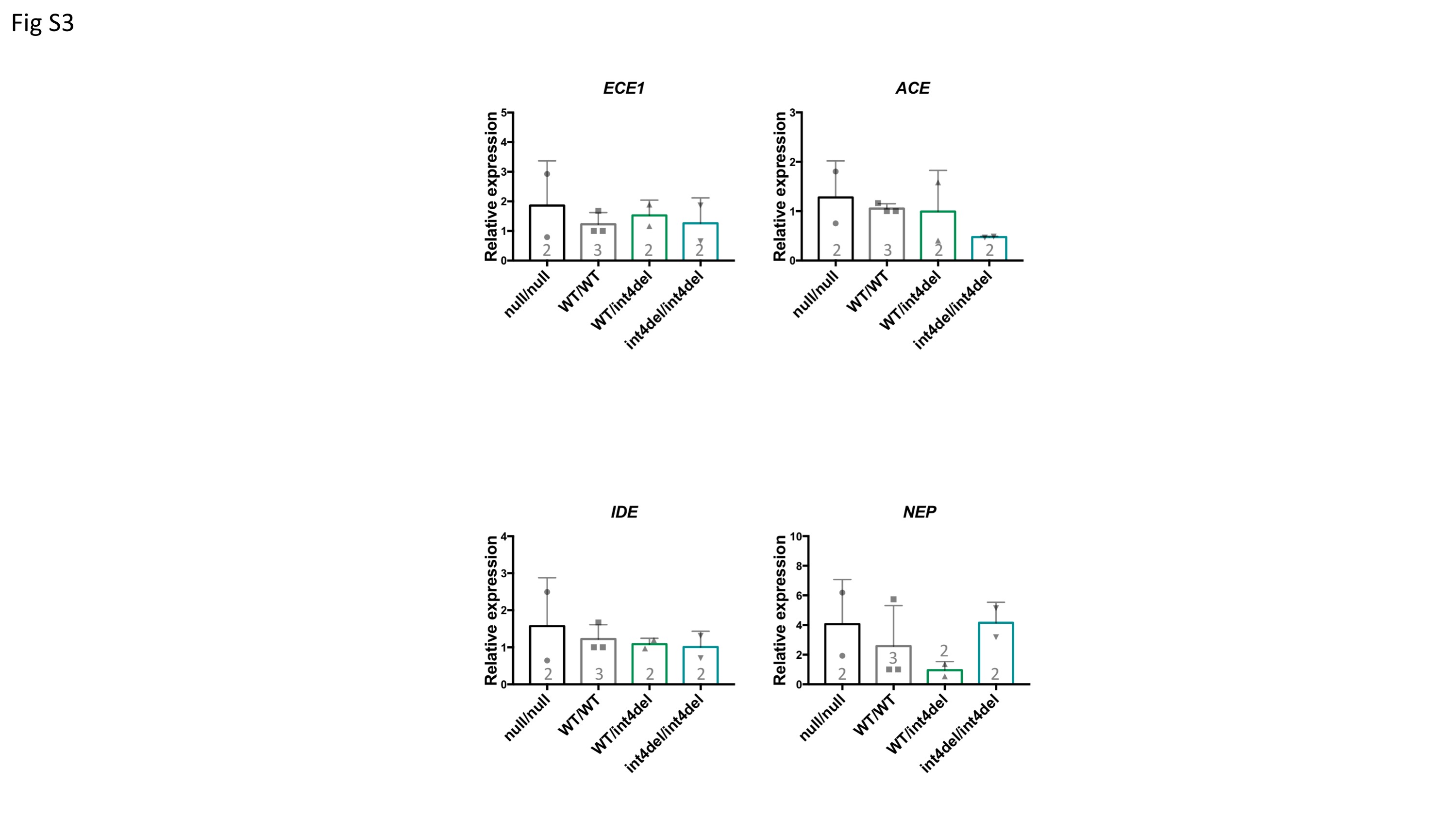

Supplement: fcz024_Supplementary_Data [file fcz024_supplementary_data.zip › Fig S3.jpeg]
